# Photovoice Revisited: Dialogue and Action as Pivotal

**DOI:** 10.1177/10497323221077300

**Published:** 2022-03-04

**Authors:** Hanna Gabrielsson, Agneta Cronqvist, Eric Asaba

**Affiliations:** 1 Faculty of Medicine and Health, School of Health Sciences, 6233 Örebro University, Örebro, Sweden; 2 Department of Health Care Sciences, Marie Cederschiöld University, Stockholm, Sweden; 3 Department of Neurobiology, Care Science and Society (NVS), Division of Occupational Therapy, 163074 Karolinska Institutet, Stockholm, Sweden; 4 Unit for Research, Education, and Development, 83294 Stockholms Sjukhem Foundation, Stockholm, Sweden

**Keywords:** photovoice methodology, narrative theory, disability, developmental disability

## Abstract

Photovoice has gained acceptance as a viable visual method to engage community members as partners in research. However, as methods associated with photovoice have developed and evolved over time, concerns have also been raised with regard to how this impacts the methodological underpinnings on which photovoice rests. The aim of this article is to explore the meaning of dialogue and action as methodologically pivotal for the relevance of photovoice as community-based participatory research; further, using an empirical case and narrative theory, we attempt to contribute to an understanding of the processes that facilitate the viability and relevance of photovoice. By unpacking the contributions of dialogue and action towards a participatory methodology, in this case photovoice, the authors illustrate and argue for aspects critical in photovoice. Drawing on these aspects provides an arena for storytelling and story making, which have not previously had an explicit part in photovoice.

## Introduction

Photovoice has gained traction as a viable method in the healthcare sciences during the last decade (Holmlund et al., 2018; Maratos et al., 2016; Mälstam et al., 2018; Ronzi et al., 2016; Watchman et al., 2020); however, in tandem with methodological development and diverse use of photovoice, concerns have also been raised with regard to rigor and critical reflection underpinning different aspects of photovoice projects (Asaba et al., 2015; Catalani & Minkler, 2010; Liebenberg, 2018; Golden, 2020). In this article, we contribute to critical reflection about how photovoice can be methodologically and technically relevant, particularly in studies that address topics at stake for persons with developmental disability and cognitive impairment. Furthermore, we will focus on the doing and dialogue in given situations, through which “voices” become audible (and visible) among people who have historically been excluded from invitations to actively be involved in participatory research. In this article, we will use dialogue and action as tools to explore narrative turning points and meaning-making embedded in an exchange of experiences enacted through photovoice sessions. An argument will be made for the importance of dialogue and action informing the entire photovoice process.

A: Here is another door, a door in a hallway.

B: Yes, right.

C: Isn’t this one, from the laundry room, isn’t this also some sort of a door?

D: Yes, keys and codes and …

E: That one …

C: Yes.

F: There you can see part of a door, right?

E: Yes!

A: That one I’d like to have, like belonging to the door as well.

C: Which one?

A: That door opener.

F: Yeah, that’s right.

C: Yes, exactly, closed and open doors we have, then.

A: Yes …

At first glance, the quote from a photovoice session lacks context and narrative meaning. However, the significance of the quote lies in the fact that it is a part of an unfolding discussion stemming from a series of photographs taken by adults living with developmental disability and cognitive impairment, as part of a photovoice research project. We will return to the above quote later in order to populate it with meaning; however, for now we use it as an illustration of dialogue, albeit at times only with a single-word remark, among a group of people engaged in a photovoice project (Gabrielsson et al., 2020).

The aim of this article is to explore the meaning of dialogue and action as methodologically pivotal for the relevance of photovoice as community-based participatory research; further, using an empirical case and narrative theory, we attempt to contribute to an understanding of the processes that facilitate the viability and relevance of photovoice.

### Background

Photovoice has been widely used since the 1990s to describe a broad range of studies from several fields applying visual methods. Many studies using photovoice have been conducted in public health or health promotion. Approximately one-fourth of these studies between the 1990s and 2010, involved individuals with chronic disease, intellectual or physical disabilities, and mental illness (Lal et al., 2012). Although methods develop and change over time, it can be important to critically reflect on the consequences and direction of development. For instance, in the case of photovoice, studies naming photovoice have not always grounded methods in conceptually important parts of community-based participatory research (CBPR) (Catalani & Minkler, 2010). Furthermore, as methods are used in different contexts, it can also be important to make visible the details of a process in order to understand its function or impact.

Photovoice has been thoroughly and eloquently described elsewhere (Wang & Burris, 1994; 1997), however, in short it is about engaging members of a given community as experts on their own life situation. Before the term “photovoice” was coined, “photonovella” was used to describe processes of using photographs or pictures to tell a story (Wang & Burris, 1994, 1997). The impetus for developing new methods to engage the community had roots in CBPR, with the objective of going beyond exploring/understanding a phenomenon and moving towards promoting social change. In tandem with the development of the concept and semantically shifting to the term “photovoice,” three main goals aligned with the methodology were put forth: “(1) to enable people to record and reflect their community’s strengths and concerns; (2) to promote critical dialogue and knowledge about important community issues through large and small group discussion of photographs; and (3) to reach policymakers” (Wang & Burris, 1997) p. 370). Photovoice can be distinguished from other methods using photos or pictures, in that members of a photovoice group actively make decisions about what photos to generate and how to generate them, as well as being active in the entire research process.

We draw inspiration from a series of critical reviews and reflections put forth, in which scholars have raised several issues such as fit of methods (Catalani & Minkler, 2010), how participant voices inform design and dissemination (Evans-Agnew & Rosemberg, 2016), connections to justice and change (Sanon et al., 2014), and ethics of equity (Golden, 2020). In 2008, the need for a systematic review of photovoice (within CBPR) was identified and presented the following year (Catalani & Minkler, 2010). The authors used a tool (Viswanathan et al., 2004) to rate the level of community participation based on 10 aspects. Studies rated as low often included minimal interaction with the researcher and/or other participants in the project, while high scores on participation were associated with ongoing partnerships with communities, longer duration, as well as an emphasis on training and community capacity building, critical dialogue, and engagement in action. The studies that were reviewed and had a medium to high participatory approach to the analysis, were found to be widely adapted to fit particular needs of research and documentation projects resulting in a broad variation on the participatory scale (Catalani & Minkler, 2010). A longstanding critique concerning the degree to which photovoice studies contribute to social change was raised in a review with focus on social justice intent, where the authors found that few studies were designed to impact on policy, and that most studies judged change to be only at the individual level (Sanon et al., 2014). A particularly surprising finding in another study was that only a relatively few number of studies use dialogue through photovoice sessions to jointly explore commonly identified topics; thus, there is some unclarity in how group members were engaged in dialogue and how discussions were used in the data analysis (Evans-Agnew & Rosemberg, 2016). Based on the findings of the most recent review (at the time of writing this paper), many of the earlier critiques continue to be identified. Golden (2020) raises four concerns, with the aim to generate dialogue about how scholars can actively continue to advance equitable research (Golden, 2020). The four concerns were (1) reported photovoice projects too rarely include participants beyond the phase of generating data, and in publications often neglect to address community outcomes; (2) ownership of materials, such as photos, or co-constructed materials, such as texts or an exhibition, is incongruent with the participatory paradigm; (3) inaccurate and unrealistic views of empowerment have led to insufficient attention to the context and meaning of how a given project has had immediate relevance for the members of the photovoice group; and (4) photovoice projects too often fail to deliver on the goal of having an impact on policy or structure (Golden, 2020).

### An Empirical Illustration

To more deeply explore and reflect on how dialogue and action is enacted in photovoice research, we use one photovoice project as an illustration. The study in its entirety has been reported elsewhere (Gabrielsson et al., 2020), however, a brief summary of relevance for this paper is presented here. The project involved four men and one woman with spina bifida (SB), who were between 30 and 49 years of age. All participants gave informed consent and ethics approval was obtained from the Regional Ethics Board in Stockholm (Dnr: 2017/992–31/2). In Sweden, SB is classified as a rare condition and is congenital involving the total central nervous system, resulting in varying levels of disability, in most cases including both physical and cognitive impairments (MMCUP, 2020). Of relevance here is to highlight a context of cognitive and physical disability as well as a history of social exclusion and isolation that persons with SB have faced for many years. There has been a common misunderstanding that persons with developmental or intellectual disabilities are incapable of expressing their own needs and of learning health-promoting skills (Jurkowski & Paul-Ward, 2007). This has traditionally led to persons with developmental disabilities, such as SB, being excluded as partners or collaborators in research projects (Ward & Trigler, 2001).

### An Overview of the Photovoice Sessions

Photovoice consisted of a series of sessions over time that served as an arena for group dialogue and action to take place and for the crafting of narratives. A brief overview of what the photovoice sessions included is presented in Table 1 (each session is represented by the number of the session and the theme that was discussed; session 1 has no theme as this was not yet determined by the group and session 8 has no theme as analyses informed the session). The structure and elements of the photovoice sessions is important to understand the progression of the project and as a foundation for understanding the methodological reflections of the analysis. At the introductory session, when the members met for the first time, ground rules were identified and agreed upon in order to create safety for the members and boundaries for the project. Members of the group raised perspectives about what was important in terms of mutual respect or for an individual person in the group. Ground rules ended up including: how the photos could be used, such as decisions not to use the photos on social media for the duration of the project; if the photo brought to the session included recognizable persons, an agreement from those persons should be ascertained. The importance of respecting each others’ stories was also agreed on, including an agreement that “what is said in the room stays in the room” until all agreed on what to do with their collective experiences. These ground rules were revisited during the project, as a reminder, and members were asked if they wanted to add or revise anything during the course of the project. This type of structural agreement serves as a way of creating common values concerning the work and partnership within a group. A similar process was described by Wallerstein et al. (2008), who also highlighted the possibility to enhance mutual respect by reflecting on core values.

**Table 1.** Sessions.

1	2 *Optional everyday life*	3 *Accessibility*	4 *Communication*
• Introduction and ice breaker • Presentation • Coffee break • Ground rules • Formulating a theme	• View and dialogue from 1st session • Coffee break • Formulating a theme	• View and dialogue photos from 2nd session • Coffee break • Formulating a theme	• View and dialogue photos from 3^rd^ session • Coffee break • Formulating a theme
5 *Rights*	6 *Experiences* (visual analysis)	7(Visual analysis)	8
• View photos shot for this session and printed from earlier sessions • Dialogue • Coffee break • Start selecting photos from all previous sessions • Formulating a theme	• View photos shot for this session and printed from earlier sessions • Dialogue • Coffee break • Selecting photos from all sessions • Group the chosen photos on the wall • Name the groups of photos	• View photos shot for this session and printed from earlier sessions • Dialogue • Coffee break • Selecting photos from all sessions • Group the chosen photos on the wall • Name the groups of photos	• In dialogue, formulating visual analysis into new themes: *Meaningful everyday life; Accessibility is a right; The struggle in everyday life; Communication* • Coffee break • Summary • Plan forward; exhibition

An important aspect of the photovoice sessions, that should not be underestimated, was the coffee breaks. The coffee breaks allowed for casual small talk and laughter interwoven with lingering topics from the session. At times, the theme of the day lingered over the break, while at other times completely different topics were discussed. Sometimes it was as if the beverages during coffee break were only a symbolic artefact that opened the door for a fellowship to grow among the group. Including a break in the session was also a way of shifting focus from the work of the project to a brief moment of rest to allow the members to collect thoughts before continuing to participate in, and reflect on, the discussions in progress.

Participatory decision making and involvement in the group was another element of high importance, for example, in deciding on the following week’s theme. In the context of conducting a photovoice project with a group that did have some degree of cognitive impairment, it was important for the facilitator to be observant and insure that every member had an opportunity to contribute. This facilitator role was also something desired and agreed upon by the group in the setting of ground rules. One way of doing this was to make sure that members took turns in sharing photos during the session and that everyone was listened to when they spoke. An important part of every session was for the group to reach agreement about a theme for the following week. If agreement could not easily be reached, that facilitator had a role to ensure that the person or persons whose perspective had not been prioritized were given priority in deciding on a theme in the next session. These ground rules were not only agreed on in the group, but were strategies that enabled each member in the group to feel included in a process even when there were slight disagreements or differences in opinions.

Other strategies for enhancing participatory decision making were to involve the members of the group in different tasks relevant for the photovoice sessions. For example, one of the members showed an interest early on in the technicalities of setting up a projector and computer. This member helped in setting up and when it was time to view photographs each week, this member took extra responsibilities for the technical parts like loading the next photo. Another member expressed that text, in addition to the images or photos, was important; this person later took extra responsibility for the task, together with one of the facilitators, to write text captions for the photos that would be included in an exhibition. Two of the members had previous experience of photography, either professionally or as a hobby, and offered to take portrait photos of the members of the group for the exhibition.

#### An Analysis and Argument for Dialogue and Action

Dialogue and action among a group of people is intimately interrelated with narrative concepts such as storytelling and story making (Clark, 1993). Storytelling or story making offers meaning, by sense making of a lived experience, and in this way is a powerful everyday tool that is further sharpened and utilized in photovoice methodologies (Clandinin, 2013). The photovoice session can provide an arena for exchange of experiences and reflections, including doing together with others. Telling a story provides opportunities to shape, and be shaped by, the narrative through which a person presents him or herself. Positioning the *word* as the essence of dialogue, inherent of both reflection and action, is pivotal in approximating ambitions for both personal and social transformation and change (Freire, 1993, p. 59). The narrative nature of people interacting through dialogue, grounded in the time span of doing, is informed by temporality, agency, and identity (Ricœur, 1984, 1985). In sessions such as those that characterize photovoice, narratives are crafted in a stream of lived experiences (Applebaum, 2015). Photovoice sessions that are orchestrated over time and with reasonable decision latitude among its group members offers opportunities to share one’s own story, listen to others’ stories, and create new stories together with others.

#### The Relevance of Dialogue

“Only dialogue, which requires critical thinking, is also capable of generating critical thinking. Without dialogue there is no communication, and without communication there can be no true education.” (Freire, 1993, pp. 65–66). Freire’s approach to learning puts the dialogue in the center of knowledge co-creation, balancing the power relations that might otherwise characterize the teacher–learner (researcher–participant) relationship. This might seem like a bold vision in relation to the opening quote, however, we argue that our introductory quote represents the beginnings of a critical perspective through dialogue. Moreover, by using both visual and verbal narrating methods, members had more than one way of conveying and communicating experiences. In the quote we used to introduce this article, the group members in a photovoice session were elaborating on chosen photos and stories that was being placed on a wall. This dialogue took place during one of the last sessions and illustrated how several members engaged.

Of relevance here is a contextual background in order to situate the opening quote. Prior to commencing this study, there had been substantial concerns expressed by external gatekeepers about involving persons with SB in this type of project because of certain cognitive impairment. During the first and second session, the researchers observed a verbal exchange among members of the group that consisted of something characteristic of a monologue by some, and conforming agreement by others. Although this intially can be perceived as challenging, establishing rapport and feeling a sense of safety to share in a group often takes time. There can be perceived imbalances in power and positioning in a group that also needs to be negotiated in order for the whole group to feel comfortable. Over time and by the eighth session, in collectively working with ground rules and through the visual analysis of grouping photos and populating the visual constellations with narrative meaning, the quote presented in the introduction of this article illustrates how the group engages together. The way in which these group dynamics developed and came to expression through a visual and qualitative analysis together exceeded expectations, and only a few months previously, the possibility of running this type of project with this group was imbued with uncertainty.

Each picture of a door had previously been discussed. The symbolic meaning originally assigned to a picture of a door based on what the “photographer” shared, was in this moment being renegotiated. For instance, was a picture of a laundry room wall in fact a picture of a door or was it something else (even if there was a glimpse of a door in the picture)? At times, the reasons for choosing a photo were mainly esthetic; at other times, the story behind the photo received more weight. Further, at the beginning, members mainly chose their own photos, but as the process evolved, several chose photos and stories that others had brought.

Dialogue is a pivotal and powerful tool in photovoice, and something that cannot be compromised in adaptations to the methods. In a review of photovoice used in disability research, it was found that the most common modification was to replace the group discussion with one-on-one interviews (Shumba & Moodley, 2018), or a combination of individual interviews and one group discussion at the end (Jurkowski & Paul-Ward, 2007). Reasons for this type of adaptation were justified in difficulties to conduct a group discussion because of individual factors, such as difficulties with concentration, or logistical factors, such as inability of all members to meet at the same time and place. Although this adaptation might be necessary in order to gather photos or to engage a particular member in an interview, it can be questioned whether this allows a project to achieve the goal of critical dialogue and the building of long-term and extended partnerships. Where individual interviews were suggested in order to limit the unwanted influence of bystanders, peers, assistants, or staff members (Overmars-Marx et al., 2018), the authors of this article challenge this view as it risks reifying the peripheralizing of voice among persons with developmental disabilities. Moreover, if the purpose is to generate photos, there are other methods available that are not necessarily guided by a participatory paradigm (Glaw et al., 2017; Harper, 2002).

#### The Narrative Significance of Dialogue

The transformation from sharing their own story with others to embracing a number of stories and beginning to rewrite/revisualize a new story was important for several reasons. The sharing of experiences among group members was at times filled with humorous expressions and at other times with frustration. This contributed to a sense of community building in which it was safe to share stories and begin playing with the exercise of rewriting the group’s evolving, co-constructed story. Initially, the narrative process may have been characterized as more performative, involving a narrator and an audience (Mattingly, 1998), but as the group grew closer, story sharing became a tool not only for sharing but also for co-creating.

The transformation is indicative of an insight that it is the group’s collective experiences, aspirations, and reflections that have made up the fabric and texture of the stories in this context. Moreover, crafting a story together is challenging and requires some degree of trust. There can be a sense of vulnerability in sharing a personal story, and it can be even more sensitive to allow another person to use, reuse, and perhaps even rewrite the narrative by abstracting certain elements. Mutual trust between members, such as in a Photovoice group, is viewed by Freire as a logical consequence when the foundation for dialogue is love, humility, and faith (Freire, 1993). By being listened to instead of “talked at,” participants in photovoice studies may perceive enhanced self-esteem and peer status, and gain a sense of political efficacy (Wang & Pies, 2008). Through the members’ active involvement in, and contribution to, the process, interactions in the group became self-sustaining and therefore encouraged more participation. A critical aspect of creating conditions for trust is the fact that there were eight group sessions, which allowed relations to develop as a base for dialogue and collective story making grounded in story sharing. This would likely have been difficult or impossible if we had used single, individual interviews or focus group interviews.

#### The Visual in Facilitating Dialogue

Conceptually working with these visual materials meant that each person put his or her photos and stories up for redefinition. This can be a challenging task, both artistically and cognitively. Through the process of working together, each member of the group, as well as the group collectively, had developed a form of literacy of raising issues of concern and transforming these into visual story plots intended for a public exhibition. Thus, the quote, which at first glance appears to lack meaning, can be seen as a representation of dialogue and literacy at the culmination of a co-creation process of planning, gathering, and analyzing data in order to put on an exhibition. It is a representation of pride in the message behind a picture and the potential power it might have, or not have, when placed in a public space to trigger dialogue about disability rights and accessibility. As stated previously, sometimes, the reason for choosing a photo was mainly esthetic and sometimes the story had more weight. At the beginning of the process, some members chose mainly their own photos, but as the process evolved, several members chose photos and stories that others had brought in. To relate to, and reflect on photos, a visual product, can be challenging and can require training for people for whom the process is new. In this group, this “visual literacy” of responding intuitively to photos was developed during the course of the project. Some authors argue for attention to the esthetic qualities of photographs (Shankar, 2016) and the potential of photovoice as an arts-based research method has been pointed out by Golden (2020). In her eloquent critique of how scholars too often detach a set of visual methods from the methodological foundations of participatory research in photovoice projects, Golden also offers suggestions for building on the method to develop more equitable and responsive research practices. In framing photovoice as an arts-based approach, for instance, there is an opening for media other than photography and also for viewing benefits in terms other than traditional health measures (Golden, 2020). The arts-based approach might risk the possibility of what an everyday photo can add to the dialogue, possible recognition and co-creation of stories, especially if members get worried that their photo is not artistic enough. Based on this project, we suggest that the photo can be part of the story. Our experiences through several projects is that the photo often can trigger metaphors among others in the group.

A debated point in photovoice has been the degree of photographic training required. Early on, photovoice was described as flexible and adaptable (Wang & Burris, 1997), making it accessible and participatory with minimal technical training. For instance, the early publications about photovoice contained reflections about whether or not to provide extensive instructions for how to take a well-composed photo versus using a photo in a more raw format to capture a moment, scene, or thought pertaining to a theme of a given project (Wang & Burris, 1997). The minimalistic training approach often means that an exhibition needs some conceptual framing for the audience to fully appreciate and understand the visual display of photos, which can be symbols of an early germination of ideas, or a powerful metaphor for an experience. The possibility to use photovoice in a flexible manner is also accompanied by a risk of losing certain essential aspects, if the techniques are to be seen as aligned with a participatory methodology and a capacity to be active in the social domain.

Other adaptations, which do not pose the same risks to a participatory design, include assistance with photographing, for instance, if the participant has visual impairments or trouble holding the camera (Shumba & Moodley, 2018). Others have suggested “guided photovoice” when involving persons with intellectual disabilities (Overmars-Marx et al., 2018), and it is proposed, with this adaptation, that individual interviews and guided photovoice walks are conducted while photographing with one member and the researcher. These examples of adaptation can be seen as technical or pragmatic solutions to enable participation. However, the type and quality of the adaptation is of relevance from the perspective of framing photovoice as a community-based participatory method, methodologically grounded in a participatory paradigm.

In the present project, the photos were seen as part of a story, equally valued as the verbally narrated part of the story, and as part of creating a space for co-creating knowledge through different ways of communicating. Even if some members in the photovoice group were used to taking photos, other members had essentially no experience with photography. Photographing every week with a specific theme in mind was a preparatory part of creating a space for dialogue. Utilizing photos adds a dimension to the situations discussed, which would not have emerged without the photo. The photographs were shot in the members’ everyday life, and thus provided insight into the everyday life of the person who took them, which was often recognizable to other members of the group. The visual image evoked thoughts among the members in the group that would not necessarily have occurred without the photo. Visual image has importance for enabling people to reflect about their community and the contradictions within it, which connects to education and critical consciousness (Freire, 1974).

### Empowerment through action and doing together

Wang and Burris (1994) view empowerment as three different processes: (1) enjoying power *to …*, as affirmative power: the ability to accomplish things; (2) having power *with …*: the ability to work with others towards a common purpose; and (3) exercising power *over …*, the ability to influence or direct others. This view of empowerment has close links with action. Action, in the context of photovoice, is often connected to the outcome and final product linked to raising awareness. Action is, however, an important element throughout the process and, apart from meaning “to take action” and emphasizing the third process of raising awareness, in this article “action” is also used in terms of accomplishing things and working with others, in coherence with the three processes in empowerment. Action in this context is the act of putting into words, both visually and through dialogue, an experience. In this process of capturing something in a picture, sharing a story about this picture, and reconstructing the meaning of what the story will come to be about, action is very much about narrativity (Mattingly, 1998).

To think out loud together, about an image or several images, is a way of co-creating meaning for chosen photos; at the same time, it constitutes visual thematization and narrative meaning-making. This form of “doing together” and being part of decision making is an important component of *action* and having a *voice*. In this sense, story making is a matter of active doing and is not a reorganization of what has taken place, but rather, as described by Vandevelde when discussing Ricoeur’s theory of narratives (Vandevelde, 2008), the making explicit of what was already implicit in action and life as potential stories. Moreover, Reed et al. (2020) suggest that engaging in everyday activities with others involves collective processes of narrative meaning making, which offers possibilities of transformation and recovery.

Shooting photos from everyday life, introduces a new sense of doing in everyday life for many photovoice group members. Bringing photos to share with others who can comment and recognize what has been photographed, share experiences that inform interpretation, or show appreciation, facilitates a doing together that has purpose. It is this purpose and openness to diverse perspectives that are given a space and place when members participate in decisions and exercise or try their voice (Wang & Pies, 2008). Formulating the *why?* of the chosen photo/story provides insight into meaning on a personal level, and allowing for a certain vulnerability that comes with having one’s work in a public domain, provides possibilities for renegotiating meaning socially.

For instance, the photos brought to the photovoice sessions potentially represented more than one topic. Through doing, such as taking photos, a three-stage process was made possible: (1) selecting; (2) contextualizing by storytelling and group discussion; and (3) thematically organizing, which is the foundation for analysis (Wang & Burris, 1997). A person’s capacity (i.e., inner physiological or psychological factors) and opportunity (i.e., external physical, psychosocial, or cultural factors), as mobilized by will, can be viewed as the person’s practical possibility for action (Nordenfelt, 2003; 2010). As described earlier in this article, members of the group had cognitive impairment (of varying degrees) in executive function, which had been raised as a concern by stakeholders outside of the research team in terms of the capacity to complete all parts of this project. However, photovoice sessions were co-created through dialogue and doing together, which can contribute to the realization of opportunities and will.

The power of doing together in this context is relevant and significant when situating the outcomes of dialogue and action in the exhibition. In the case of this photovoice project, the stories and photos generated by the group impacted on communities outside the group through an exhibition. Local politicians and members of patient organizations were invited to the opening of the first exhibition, and participated in round table conversations and debates about issues from the exhibition. The members of the group were actively involved in suggesting venues for the exhibition, resulting in 10 separate exhibition locations thus far.

It is difficult to measure social change, and social change requires time, often beyond the reach of a research project. As has been argued elsewhere (Sanon et al., 2014; Golden, 2020), photovoice projects often fall short of demonstrating or reporting social change. Important to note here is that the exhibition has traveled across national borders with members of the group, even though the research project had reached an end. Although not a measure of social change, it can be argued that the perservation and continued engagement in sharing their story as well as the continued interest in inviting this story, is an indication of the potentiality of social change.

## Summary

We have illustrated and argued for how dialogue and action can be enacted in the context of photovoice. We have also used narrative as a tool to illustrate the importance and relevance of dialogue and action. Although we have not had the ambition to provide step-by-step recommendations in the how-to’s of photovoice in this article, we have intended to contribute with vital conceptual tools with which to design photovoice to be robust in data generation and initial analysis. The potential merits of photovoice rests on facilitating real opportunities for members of the group to be involved in dialogue and action grounded in everyday life and participation, which will be unique in each project thus making concepts and the reasoning approach important beyond the practical setting up of a project. If through photovoice, dialogue and action are part of creating and co-creating stories about insider perspectives that highlight social inequities, injustices, or simply ignorance, then the potential for change through collective action and community engagement can emerge.
